# Hospital and Institutional News

**Published:** 1920-11-27

**Authors:** 


### HOSPITAL AND INSTITUTIONAL NEWS.

### THE MEDICAL "ADVISERS."

We note that during the week *The Times* has drawn critical attention to the Ministry of Health's appointment of a number of doctors who are to act as "advisers" in a corresponding number of "regions" into which the country has been divided. This is hardly a fresh piece of news and our contemporary's object in thus suddenly recapitulating the facts appears to be that of making a comparison between these appointments and what should have been the logical first step if Lord Dawson's scheme was about to be developed. Certainly, if Dr. Addison's thirty-four medical "advisers" are supposed to represent a beginning of the Consultative Council's suggested unit (primary health centres) system for the whole country, then we think that, from many points of view, the procedure is singularly unsatisfactory. But it appears that the new appointments represent an attempt to improve the insurance medical service as such, and the above comparison cannot then in fairness be drawn. There is a world of difference between the practice of the voluntary hospitals, non-insurance practice, and the service to which these "advisers" definitely belong, and although it is undoubtedly correct to draw public attention to any innovation which represents further monetary expenditure by the Government departments, we think that the degree of contrast which our contemporary has attempted in this instance to picture is hardly justifiable in view of the true sphere of the newcomers' duties.

### THE PARLIAMENTARY HOSPITAL MEETING.

As an outcome of the meeting of members of both Houses of Parliament, who mostly were interested in, or actively associated with, voluntary hospitals, Colonel F. B. Mildmay, who occupied the chair, has addressed a remarkably able letter to the Prime Minister informing him of the outcome of the deliberations. The formal resolution will be found on page 186, but the letter enlarges considerably upon it. While recognising that legislative reconsideration of the whole question is immediately necessary, the meeting does not consider that Clause 11 of the Ministry of Health Bill goes to the root of the matter, apart from the many serious objections to the clause. Time must be given to gather and co-ordinate the considered opinions, founded on long experience, of medical authorities, of hospital boards, of hospital staffs, and of others whose views would be valuable. A sound and lasting solution, Colonel Mildmay suggests, can only result if such opinion be available to form the foundation of legislative action. The British Hospitals Association is suggested as a good agency for the collection of the necessary data. As to the composition of the Committee of Inquiry, it is suggested that members should be quite few in numbers, and that, "in the light of recent experiences in connection with committees of inquiry, they should be absolutely impartial. Medical authorities, however eminent, and representatives of particular hospitals, should not be included, while the valuable evidence to be furnished by these should be subjected to attentive and exhaustive examination by the committee." Granted that the financial position of the hospitals worst hit is temporarily alleviated, it is thought that no harm can come of delay in legislation during the month or two that the Committee of Inquiry will take to present its report.

### ANOTHER SCHEME BY MR. COLES.

The scheme suggested by Mr. R. J. Coles, the Secretary of King's College Hospital, in which persons insured under the National Health Act are given an opportunity of assuring institutional treatment by the payment of 2d. per week, is now added to by its author to enable those who are not insured to come in. The great difficulty, as Mr. Coles is ready to see, is the collection of these small sums, but he thinks this might be brought about by the issue of hospital tickets on the 'buses and trams or by fixing attractive collection boxes in vehicles. The latter plan, we are told, was successfully carried out in Bristol some years ago for the benefit of the Royal Infirmary.

### LIVERPOOL CORPORATION AND THE ROYAL INFIRMARY.

Speaking in the House of Commons on November 4 on the second reading of the Ministry of Health (Miscellaneous Provisions) Bill, Dr. Addison said: "Since 1916 various authorities have taken power by Private Bills in this House to acquire authority to make these contributions to hospitals. There have been four cases before the House in the last twelve months. The city of Liverpool has taken power to contribute £5,000 yearly to the Royal Infirmary." Mr. W. Rutter, General Superintendent and Secretary of the Royal Infirmary, says that he is unable to get any confirmation of this statement locally, and assures us definitely that the Infirmary does not receive the £5,000 a year. He asks us to verify the quotation, which we do, and to give publicity to his remarks, as he fears that if it is thought that a new £5,000 a year is coming in it is likely to affect voluntary contributions.

### ATMOSPHERIC POLLUTION.

The sixth annual report of the Advisory Committee on Atmospheric Pollution is in parts somewhat technical, but throughout most interesting reading to those directly concerned with general health questions. In the first place, it is good to learn that a specially organised department under the Meteorological Office has considerably advanced both the methods of determining the kind and quantity of polluting substances and the schemes of attack against them. Also the total solids deposited at practically all the twenty-nine stations considered have diminished during the 1919-20 season as compared with the previous year. It is suggested that this is partly due to the increased use of gaseous fuel, and these investigations have shown a fixed cycle in the distribution of solid impurities throughout the twenty-four hours which still brings down a definite reproach upon the open coal fire. At 6 A.M. when the fires are lighted, impurity steadily increases up to about 11 A.M., and remains nearly stationary until 10 P.M., after which it rapidly diminishes again and has almost disappeared by midnight. Another question discussed is the acidity of town air, and its destructive qualities, and the degree to which the coal fire is to blame. But on this point no certain figures are so far available, and it seems open to doubt whether the domestic grate is a serious offender in this respect. At first sight, these matters appear to be relatively simple problems, but, after reading the committee's report, one realises that the simplicity lies only in the obviousness of the benefits to be derived from a cleaner town atmosphere. Investigational work in this direction should prove invaluable.

### MR. OATLEY'S SCHEME FOR NORTHAMPTON.

The Northampton General Hospital has been considering a comprehensive scheme of alterations and improvements, which it is hoped to carry out piecemeal as occasion permits. The author of the scheme is Mr. Oatley, of the London Hospital, who was engaged to advise the committee. His plans, which include a new nurses' home, a theatre block, isolation wards, kitchens, casualty department, and mortuary, together with central heating, would cost £116,000. Mr. Oatley's fee is two hundred guineas, and the committee will pay his travelling expenses. The new heating and power station will be begun promptly. Not only is it urgently needed, but a grant of £8,000 from the Red Cross is conditional on the work being begun before December 31.

### EX-SERVICE MEN'S CARNIVAL AND EXHIBITION

Under the auspices of Field-Marshal Earl Haig, Admiral of the Fleet Earl Beatty, the Marquis of Crewe, Lord Lonsdale, Air-Marshal Sir H. M. Trenchard, the Lord Mayor of London, Kathleen Viscountess Falmouth, and an influential committee representing the National Federation of Discharged and Demobilised Soldiers and Sailors, Comrades of the Great War and the Officers' Association, an ex-Service Men's Carnival and Industrial Exhibition will be held at the White City during the Christmas Holiday season. A portion of the main building, which has been granted by Mr. Charles Kiralfy, will be set aside for a demonstration of the possibilities of the employment of ex-Service men in commerce and industry by means of an exhibition of the results of their labours in various arts and crafts. Leading British firms of every branch of industry have patriotically promised to assist in making the Exhibition worthy of the occasion, and an Employment Bureau under the auspices of the Ministry of Labour, the Education Department, the Ministry of Pensions and other ex-Service men's welfare agencies, will be run, thus bringing the capabilities of skilled and unskilled to the notice of employers and placing them in touch one with the other. Various entertainments, including military bands, concerts, cinemas, a miniature circus, Christy Minstrels, billiard tournaments, ex-Service men's boxing competitions and brass band competitions, will be presented daily, while Divisional Concert Parties will repeat performances as given at the Front. On Christmas Eve, Boxing Night and New Year's Eve, plain and fancy dress balls will be given, and the attractions of the "Children's Section" will include a huge Christmas Tree and a Bazaar stocked with toys of ex-Service men's manufacture.

### A NOTABLE APPOINTMENT.

We learn that Professor H. MacLean has been appointed Director of the Clinical Medical Unit at St. Thomas's Hospital, and the announcement gives yet another indication that there is to be a fuller recognition of the direct value of the scientist, here a physiologist and biochemist, in the advancement of clinical medicine. Dr. MacLean graduated M.D. at Aberdeen in 1914. In 1912 he became D.Sc. (Lond.), and previously in 1910 was awarded M.Sc. (Liverpool). At the present time he is Professor of Chemical Pathology in the University of London, and was formerly connected with the Lister Institute. The placing of a man of such wide scientific experience in control of a clinical unit which, though with an essentially teaching objective, has also its obvious connection with future research in the true sense, is a definite indication that some at least of the principles referred to on p. 192 are realised and appreciated.

### THE LEAGUE OF MERCY AND THE "HOSPITAL CRISIS."

The Executive Council of the League of Mercy, of which His Royal Highness the Prince of Wales is Grand President, and which was established in 1899 by King Edward to enlist the sympathy and support of all classes of the community in the work of the voluntary hospitals, desire to call public attention to the facilities which its organisation affords to those who are willing to render aid to the London and provincial hospitals at the present time, when the heavy calls upon their services demand more generous support from the public if they are to be saved from state or municipal control. The League during the last twenty years has collected and distributed to voluntary hospitals in London and the provinces more than £300,000, and has an organisation at work in every metropolitan borough and in nearly every provincial district. All information may be obtained on application to the Secretary at 29 Southampton Street, Strand.

### THE PRIME MINISTER HELPS THE LIMBLESS.

The principal of an interesting series of musical events on Saturday, December 4, in aid of the Prince of Wales' Hospital for Limbless Sailors and Soldiers, Cardiff, is a Male-voice Choir Competition, which is likely to bring a record entry owing to the value of the first prize, a gold cup presented by the Right Hon. D. Lloyd George, Prime Minister, together with £100 contributed by a friend. This competition will take place in the Empire Theatre, Cardiff (seating over 3,000 people), and will last from 11 o'clock A.M. until about 5 P.M. The Committee has been fortunate in obtaining the services of Professor H. Walford Davies, Mus.Doc., LL.D., F.R.C.O., as adjudicator. The second prize is £50. Simultaneously with this competition there will be taking place in the Assembly Room of the City Hall, Cardiff, preliminary tests for two Champion Solo Competitions, one open to males and one to females, the first prize in each case being £10. As the competitors are free to sing whatever selection they choose, the competition is likely to be interesting. In this case the adjudicator is Mr. Ivor Atkins, Mus.Bac., F.R.C.O., organist of Worcester Cathedral and Conductor of the Worcester Festival. The Welsh are a musical race, and the competitions should attract keen attention.

### A HOSPITAL MOVES.

ON November 18 the staff and patients of the Alexandra Hospital for Children Suffering from Hip Disease moved by road from its familiar quarters in Queen Square, Bloomsbury, to a temporary house at Swanley, Kent. The London site has been sold to a gas company, which proposes to convert it into offices, with very little modification. The move was accomplished in motor-ambulances and twenty pantechnicons. The travellers included fifty-two patients and thirty nurses.

### RAMSGATE AFTER THE WAR.

A GOOD deal of sympathy is excited by the condition of Ramsgate General Hospital, which has revised its rules and determined on a charge of two shillings a day towards the cost of the patients' maintenance. Ramsgate was virtually provided with a hospital by the generosity of the late Mr. John Nicholas, who bequeathed £16,000 for the purpose. A band of loyal subscribers has been constant in support, but the original members have naturally been thinned by time and change. During the war, like other East Coast towns, Ramsgate suffered, but since the conclusion of peace the number of residents has risen very greatly. These have added to the hospital's patients: they have hardly added at all to the hospital's funds. This is a serious matter. Mr. C. Wilks, the chairman of the hospital's committee, has been explaining the reason for the patients' payments. We hope that some public steps will be taken to bring home to the new residents at Ramsgate their duty to the hospital of the town.

### EX-WAR HOSPITAL DESTROYED BY FIRE.

CARLOGIE HOUSE, Carnoustie, one of the best-known houses near Dundee, has been burnt to the ground by a disastrous fire, which was caused apparently by an explosion in the gas plant during the course of some alterations to the house. During the war the Countess of Dalhousie placed Carlogie House in the hands of the Carnoustie Red Cross Association for use as an auxiliary hospital. It became one of Scotland's best-known convalescent institutions. Only a few weeks ago the house was sold to a private tenant, who had begun to place his furniture in the house at the time of the fire. The damage is estimated to be £15,000. Standing on the slope above Carnoustie, the burning house was visible for two miles, and, in spite of the efforts of the Dundee Fire Brigade, was completely destroyed.

### A MODEL OF PROSPERITY.

THE institution known as Longton Cottage Hospital, Staffordshire, seems to have outgrown its modest title, and is altogether a very busy and prosperous hospital. The balance in hand rose by £200 during the past year, and now amounts to practically £900. This is remarkable in itself, especially since the Longton Hospital Saturday Fund gave no subscription, because the Committee of the fund is busy raising £1,000 for some special need at the institution. Already of this over £800 is in hand. The workpeople's contributions continue to grow, and the hospital is generously provided with all that it requires of coal and crockery. It has never had to pay a penny for coal. Mr. W. H. Jones, the secretary, has made the most of these advantages, and the present fortunate position of the hospital is due largely to him. Mr. George Nixon, who has been connected with the hospital for years, and long before the present buildings were erected, is retiring from the directorate because of ill-health. His has been a fine record of work and devotion. The Duke of Sutherland has promised to give a parcel of land adjoining the hospital, which can be used for extension. The conveyance is not yet complete. Last year the number of in-patients rose from 350 to 422, and this "cottage" hospital treated 7,380 out-patients, or almost six times as many as in the preceding year. Wherever one looks signs of prosperity and activity are seen, and one wonders if a more prosperous voluntary hospital is in existence.

### CHILD VICTIMS OF VENEREAL DISEASES.

RECENTLY has been published an influentially signed appeal for the Children's Rescue Fund, of which Lady St. Helier is chairman. For some time this organisation has made provision for the care of children infected with venereal disease, but hitherto no institution has been available for the specific purpose of treatment and after-care. The Federation of Children's Rescue Committees have now secured Coldharbour House, Waddon, Surrey, for use as a medical home, which will thus provide a building standing in its own grounds with a large garden for the children to enjoy. The hon. treasurer is Mr. R. Davies-Colley, M.Ch., F.R.C.S., 10 Devonshire Place, W. 1. In connection with this appeal, Mr. H. J. Eason, the secretary of the London Lock Hospital, Harrow Road, reminds us that his institution has been carrying on identical work for a long time.

### WELSH TUBERCULOSIS CHAIR.

THIS appointment has been filled in the person of Colonel Lyle Cummings, lately Professor of Pathology at the military hospital at Millbank. The new tuberculosis professor qualified in 1896, and apparently immediately entered the R.A.M.C., where he has been ever since. His interest in tuberculosis has not been apparent until quite lately, an article of his, on tuberculosis in uncivilised communities, being published last September. The only other British chair of tuberculosis is, of course, that at Edinburgh, filled by Sir Robert Philip, a justly famed tuberculosis clinician. We notice in the *Lancet* an annotation pointing out the danger of confining tuberculosis study, as has been too much the case in the past, to pathology and bacteriology. Our contemporary even opines that for the last forty years we have become, in looking down microscopes at "blobs of carbol fuchsin," even as the hen whose beak has been laid on a chalk line. This criticism is no doubt just and timely. All things considered, the result of this election—we do not know who the other candidates may have been—seems a strange one.

### BOURNEMOUTH'S CHRISTMAS-BOX.

A special endeavour is being made at Bournemouth by the Hospital Saturday and Sunday Fund to hold a general collection during Christmas week on behalf of the hospitals. The industry of Bournemouth is its visitors, and these birds of passage are proverbially difficult to organise. Hotels and boarding houses are to be asked to make a collection during the Christmas dinner, a festival which is increasingly being held away from home. The clubs, institutes, and the municipal orchestra are likewise being enrolled, and on Saturday, December 11, a flag-day collection will be made by the ladies of the town. We hope that the result will be successful, and that the week's endeavour will become an annual one. We have often advocated systematic attempts in holiday resorts, like Bournemouth, during the Christmas and summer seasons. Some public-spirited person has first to be found, and we are glad that one has now appeared at Bournemouth.

### THE COURTAULD AND HALSTEAD COTTAGE HOSPITAL.

The committee of Halstead Cottage Hospital, which was founded by the late Mr. George Courtauld, has received a generous gift of £4,000 from his daughter, Dr. Elizabeth Courtauld. Mr. Courtauld was a generous founder, and gave to the hospital two adjoining houses and adjacent land. The new building which is now possible will form an out-patient block with special departments, and become, it is hoped, a child welfare and general "preliminary centre" for the district. Mr. W. T. Cressall, of Colchester, is the architect, and the balance, after the expenditure of £2,790 on the new building, will be used to instal an *x*-ray equipment. This is another fine example of a family's interest in a voluntary hospital continued from one generation to another.

### THE POSITION AT CARDIFF.

The result to date of the Lord Mayor's appeal on behalf of King Edward VII. Hospital, Cardiff, is £120,000, or four-fifths of the amount asked for. A final effort is being made, and Sir Napier Burnett's recent visit, when he attended a conference called by the emergency and reference committee to consider the situation, once more focussed attention on the institution's needs. He again insisted that the customary method of a general appeal must be supplemented by more assured means of income. He remarked that the commercial community of Cardiff was not doing its duty by the hospital, and his familiar argument for co-ordination between all the institutions in the country included a plea that the municipality should stand by the hospital in its present difficulty, if only because every kind of case and accident is dumped at the hospitals' doors. Sir Napier's plan will reduce the waiting-list and make more economic use of the hospital beds in the county.

### COMBINED DUTIES FOR NURSES.

The Essex County Council for some time has had under consideration a combined scheme of maternity and child welfare and school medical service. Now the Ministry of Health intimates that it is of opinion that it would be preferable that the nurses appointed to undertake the combined duties in the several areas should be employed and paid by the County Council, and suggests that the County Council should apply for an order transferring the administration of the Act to the various districts affected.

### MIDWIVES ON COMMITTEES.

The South-East London Midwives' Association has passed a resolution asking the Deptford Borough Council to grant representation to the Association on the Maternity and Child Welfare Sub-Committee in order that, through the Association representative, it may take part in the health work of the borough. It may be mentioned that local authorities are empowered to appoint on Maternity Committees "persons specially qualified by training and experience in subjects relating to health and maternity."

### THIS WEEK'S DRUG MARKET.

The downward movement of prices has become accentuated, but in the absence of demand it is difficult to say what prices are in some cases, and sellers who are anxious to liquidate their holdings would accept something below current quotations. Quinine is more plentiful and the price has a slightly lower tendency. Phenacetin, phenazone, and most of the synthetic drugs are again rather cheaper, and the demand is extremely dull. Liquorice juice is offered at substantially lower rates. Cocoa butter, chloral hydrate, sugar of milk, lemon oil, bromides, and balsam tolu have again moved downwards in price. At the public auction of drugs recently held in Mincing Lane imports that had accumulated during the past three months were offered, the supplies being the largest offered at the periodical drug sales for many years. The demand was extremely poor, and the great bulk of the offerings failed to find buyers; for the most part lower bids were accepted where sales were effected.

